# Oxford Nanopore MinION Sequencing Enables Rapid Whole Genome Assembly of *Rickettsia typhi* in a Resource-Limited Setting

**DOI:** 10.4269/ajtmh.19-0383

**Published:** 2019-12-09

**Authors:** Ivo Elliott, Elizabeth M. Batty, Damien Ming, Matthew T. Robinson, Pruksa Nawtaisong, Mariateresa de Cesare, Paul N. Newton, Rory Bowden

**Affiliations:** 1 Lao-Oxford-Mahosot Hospital-Wellcome Trust Research Unit, Microbiology Laboratory, Mahosot Hospital, Vientiane, Lao PDR;; 2 Nuffield Department of Medicine, Centre for Tropical Medicine and Global Health, University of Oxford, Oxford, United Kingdom;; 3 Wellcome Centre for Human Genetics, University of Oxford, Oxford, United Kingdom;; 4 Mahidol-Oxford Tropical Medicine Research Unit, Faculty of Tropical Medicine, Mahidol University, Bangkok, Thailand

## Abstract

The infrastructure challenges and costs of next-generation sequencing have been largely overcome, for many sequencing applications, by Oxford Nanopore Technologies’ portable MinION sequencer. However, the question remains open whether MinION-based bacterial whole genome sequencing is by itself sufficient for the accurate assessment of phylogenetic and epidemiological relationships between isolates and whether such tasks can be undertaken in resource-limited settings. To investigate this question, we sequenced the genome of an isolate of *Rickettsia typhi*, an important and neglected cause of fever across much of the tropics and subtropics, for which only three genomic sequences previously existed. We prepared and sequenced libraries on a MinION in Vientiane, Lao PDR, using v9.5 chemistry, and in parallel, we sequenced the same isolate on the Illumina platform in a genomics laboratory in the United Kingdom. The MinION sequence reads yielded a single contiguous assembly, in which the addition of Illumina data revealed 226 base-substitution and 5,856 indel errors. The combined assembly represents the first complete genome sequence of a human *R. typhi* isolate collected in the last 50 years and differed from the genomes of existing strains collected over a 90-year time period at very few sites, with no rearrangements. Filtering based on the known error profile of MinION data improved the accuracy of the nanopore-only assembly. However, the frequency of false-positive errors remained greater than true sequence divergence from recorded sequences. Although nanopore-only sequencing cannot yet recover phylogenetic signals in *R. typhi*, such an approach may be applicable for more diverse organisms.

## INTRODUCTION

Until recently, whole genome sequencing (WGS) has been the preserve of high-income settings. Although the costs of WGS have dramatically decreased over the past decade,^1^ the need for initial investment in sequencing platforms and associated equipment and for the technical expertise to run and maintain them have prevented their introduction into lower income settings. Downstream processing of sequencing data is frequently hampered by poor Internet connectivity, a lack of local expertise, and the perceived requirement for substantial computational infrastructure.

Oxford Nanopore Technologies’ (ONT) MinION sequencer is a portable device for DNA and RNA sequencing that generates data for local analysis, in real time. The MinION weighs under 100 g and plugs directly into a laptop via a USB port, with no additional computing infrastructure required.^2^ Only basic laboratory facilities are needed to extract DNA and prepare sequencing libraries. The MinION has been used to sequence viral,^3^ bacterial,^4,5^ and eukaryotic^6–9^ genomes. Despite its high raw error rate, nanopore data can in many cases produce highly contiguous assemblies. Illumina short read data can be added to improve consensus accuracy, in many cases to that of a high-quality draft assembly, without extra finishing steps.

The Lao People’s Democratic Republic (Laos) is a land-linked country of ∼7 million people in Southeast Asia. Lao People’s Democratic Republic is ranked 135th in the world on the Human Development Index, a composite statistic of life expectancy, education, and per capita income.^10^ There are few functioning molecular biology laboratories in Laos, no previous published WGS projects, and relatively poor Internet connectivity.^11^

*Rickettsia typhi* is an obligate intracellular, Gram-negative bacterium in the family Rickettsiaceae that causes the disease murine typhus and has a worldwide tropical and subtropical distribution.^12,13^ The pathogen is transmitted to humans primarily by flea fecal contamination of the bite of the oriental rat flea *Xenopsylla cheopis*.^14^ The disease is an important and grossly under-recognized global cause of febrile illness.^15^ Those affected suffer from high fever, headache, myalgia, arthralgia, nausea, and vomiting and may have a macular rash. Complications are infrequent but include myocarditis, meningoencephalitis, seizures, and renal failure.^13,16^ When diagnosed, recovery is typically rapid after treatment with doxycycline and mortality is low (around 1–2%) with antibiotic treatment.^12,17^

Despite its importance as a pathogen, to date just three *R. typhi* whole genome sequences, with wide geographic and temporal distribution, have been published. The type strain Wilmington was isolated from a patient in North Carolina in 1928, and the sequence was published in 2004.^18,19^ Two further sequences originating from a patient in Northern Thailand collected in 1965 and the other from a bandicoot rat, *Bandicota* sp., collected in Burma (now Myanmar) in the 1970s^20–22^[Bibr b20][Bibr b22] were published online in 2014 (Table 1).

**Table 1:** Strain information

Strain	Source	Accession	Genome length (bp)	Predicted genes	GC percentage
Wilmington	Human, North Carolina, USA. 1928	NC_006142.1	1,111,496	865	0.289
B9991CWPP	Bandicoot rat, Myanmar. 1970	NC_017062.1	1,112,957	865	0.289
TH1527	Human, Chiang Rai, Thailand. 1965	NC_017066.1	1,112,372	865	0.289
TM2540	Human, Bolikhamxay, Laos. 2012	ERZ497871	1,111,939	866	0.289

The Wilmington, B991CWPP, and TH1527 strains are previously assembled strains of *Rickettsia typhi*; TM2540 is the assembly produced in this study.

We report the successful WGS of a human isolate of *R. typhi* using only the MinION platform in Laos. We assess the potential for use of ONT data alone to perform comparative analyses and discuss the challenges of undertaking WGS in resource-limited settings.

## MATERIALS AND METHODS

### *Rickettsia typhi* culture and DNA extraction.

Frozen L929 mouse fibroblast cells (ATCC CCL-1), infected with *R. typhi* isolated from the blood of a patient, TM2540, from Pakkading district (∼18.33°N 104.0°E), Bolikhamxay Province, Laos, with suspected acute murine typhus infection presenting in 2012 to Mahosot Hospital, Vientiane, were reanimated. Frozen aliquots were briefly thawed at room temperature and then transferred to 25-cm^2^ cell culture flasks containing an L929 cell monolayer at 80% confluence in RPMI 1640 medium (Gibco, Thermo Fisher, Waltham, MA), supplemented with 10% fetal calf serum (Sigma-Aldrich, St. Louis, MO). Flasks were incubated at 35°C in 5% CO_2_ atmosphere for 7 days, and a fraction of cells were mechanically detached and 3 mL transferred to a new 75-cm^2^ flask containing an L929 monolayer and incubated for a further 7 days in the same conditions.

For DNA extraction, cells from three 75-cm^2^ flasks were mechanically detached, resuspended, and transferred to a 50-mL conical-bottom centrifuge tube and centrifuged at 3,220 × g for 10 minutes. The pellet was resuspended in 3-mL fresh medium and transferred to 1.5 mL microcentrifuge tubes. Tubes were vortexed for one minute and centrifuged at 300 × g for 3 minutes. The supernatant was passed through a 2-µm filter (Corning, Corning, NY), and DNAase was added to the filtrate to a final concentration of 14 µg/mL and, then, incubated at room temperature for 30 minutes. The mixture was centrifuged at 18,188 × g for 10 minutes, and the pellet washed twice with 0.3 M sucrose (Sigma-Aldrich). Then, DNA was extracted from the *R. typhi* pellet using the DNeasy Blood & Tissue kit (Qiagen, Hilden, Germany) following the manufacturer’s protocol. Then, DNA was eluted in AE buffer and stored immediately at −20°C.

The eluted DNA was quantified using Qubit dsDNA High Sensitivity assay kit (Thermofisher, Waltham, MA) following the manufacturer’s protocols and assayed for *R. typhi* sequences by the quantitative polymerase chain reaction targeting the 17-kDa outer membrane antigen.^23^

### Preparation of MinION sequencing libraries.

The MinION sequencing library was produced using the ONT 1D genomic DNA by following the ligation (SQK-LSK108) protocol. In brief, 1.4 µg of DNA was fragmented in a Covaris g-TUBE (Covaris Ltd., Brighton, United Kingdom) by centrifugation to produce fragments of 8 kb in length. Sheared DNA was repaired using NEBNext FFPE repair mix (New England Biolabs, Ipswich, MA). End-repair and dA-tailing were performed with the NEBNext Ultra II End Repair/dA-Tailing module. Adapter ligation used the NEB Blunt/TA Ligase Master Mix, and the library was purified using Agencourt AMPure XP beads (Beckman Coulter Inc., Brea, CA).

### MinION sequencing in Laos.

MinION libraries were sequenced for 48 hours on an ONT MinION R9.5 flow cell, connected to a Dell Latitude E5470 XCTO laptop with 256 GB SATA Class 20 solid state drive. Oxford Nanopore Technologies fast5 data files were base-called using the ONT Albacore module v2.2.7.

### Illumina sequencing in Oxford.

An Illumina sequencing library was generated from the same sample of *R. typhi* DNA using the Nextera XT (Illumina) library preparation method. The library was sequenced on the Illumina MiSeq with 2 × 250 bp reads. A total of 2,402,136 read pairs were sequenced, giving 1.2 Gbp of total sequence data.

### Bioinformatic analysis.

The species composition of the MinION reads was identified using Centrifuge v1.0.3 software^24^ testing against the prebuilt nonredundant database and keeping only the best hit per read.

Assembly strategies that used MinION data alone, or in combination with Illumina short reads, were assessed. A draft genome assembly was generated from the MinION reads using Canu v1.4^25^ with the suggested parameters for ONT sequencing “correctedErrorRate = 0.120 -nanopore-raw” and an estimated genome size of 1.1 Mb and polished using Nanopolish v0.10.2.^4^ The Illumina reads were mapped to the Canu+Nanopolish assembly using bwa 0.7.12,^26^ and the mapped reads were used to error-correct the assembly using Pilon v1.22^27^. To check for enrichment of errors in regions of low coverage, the ONT reads were mapped onto the corrected assembly using bwa mem to determine coverage, and the coverage at error positions was compared with the total coverage distribution. To look for enrichment of errors near homopolymers, we compared the distance with the nearest homopolymer of 5 bp or longer in length for each corrected error to a random sample of positions in the genome. To assess the gene content, BUSCO (Benchmarking Universal Single-Copy Orthologs) v3 using the OrthoDB v9 bacterial data set was used.^28^ The Illumina short reads alone were assembled using Unicycler.^29^ Prokka v1.14^30^ was used to annotate both the new assemblies and the existing *R. typhi* genomes to give consistent data for comparison. The Wilmington reference strain was used to train the gene model for the Prodigal gene prediction used in Prokka, and this training set was used to annotate the other three genomes. The new genome was rotated to begin with the *yqfL* gene for consistency with other *R. typhi* genomes. Roary v3.12.0 was used to determine the core and accessory genomes.^31^

### Data availability.

The three existing *R. typhi* genomes were downloaded from RefSeq under the accession numbers NC_006142.1, NC_017066.1, and NC_017062.1. Gene identifiers are given as the locus tags in these annotations. The sequence data are available at the ENA under project PRJEB2567. The new strain is named TM2540, and the assembly (final error-corrected version) is available at the ENA under accession number ERZ497871.

## RESULTS

The MinION flow cell and reagents were shipped from Oxford, United Kingdom, to Laos at +4°C. Flow cell quality control before dispatch recorded 1,242 active pores and on receipt after 72hours travel, 1,103 active pores were recorded as available. Sequencing using the MinKNOW platform was performed for 48 hours and generated approximately 250,000 reads, with a total fast5 file size of 20 GB. In total, 222,848 reads passed quality filters and were used in further analysis.

**Figure 1.**
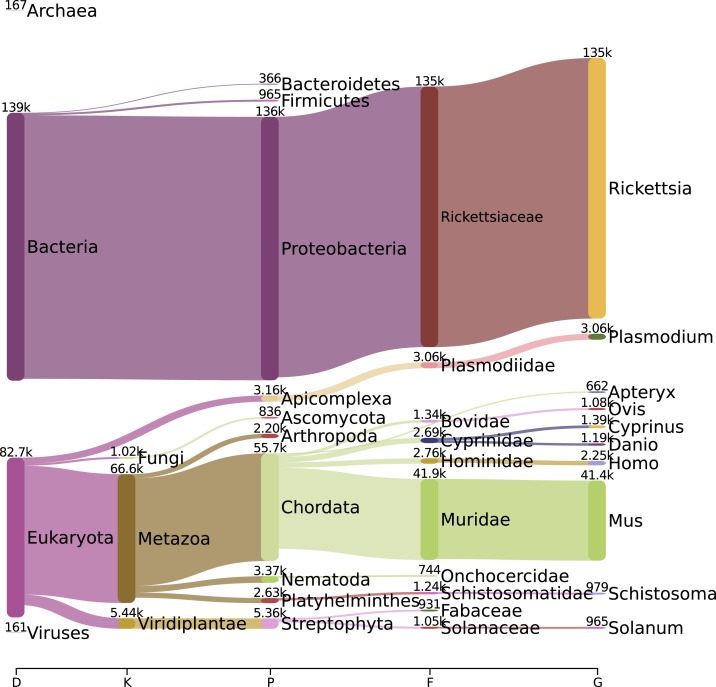
Pavian visualization showing the proportions of Oxford Nanopore Technologies′ reads assigned up to the genus level^32^. This figure appears in color at www.ajtmh.org.

We assessed the species composition of the reads that passed quality filters. Because the *R. typhi* sample was grown in L929 fibroblast cells, we expected to see some contaminating reads from the mouse genome. In all, 134,802 (60%) of the reads were classified as belonging to the genus *Rickettsia*, 126,447 reads were classified as *R. typhi* at the species level, and 41,428 (19%) were classified as belonging to the genus *Mus* (Figure 1).

The FASTQ files produced by Albacore were assembled using Canu, an assembler designed for noisy single-molecule sequencing such as that produced by the MinION. This produced a single-contig assembly of 1,078,916 bp. Nanopolish, a tool to use the signal-level data from Oxford Nanopore sequencing, was used to improve the consensus sequence of the Canu assembly, producing a new assembly of 1,099,322 bp. To further correct errors in the assembly produced from long reads alone, we polished the Canu + Nanopolish genome assembly using Pilon, which uses short read sequencing data to correct errors. We repeated rounds of mapping and correction with Pilon until no further errors were corrected. After four rounds of correction, Pilon did not correct any further errors, with the exception of two short indels which were removed and then reinserted by successive rounds of polishing. In total, 94.2% of errors were corrected by the first round of polishing, with a further 4.8% corrected in the second round (Supplemental Table 1).

After this process, a total of 6,082 errors were corrected, with over 95% (5,856) being small insertions and deletions, with the result that 12 kb was added to the genome during polishing for a final length of 1,111,939 bp. In comparison, when Pilon was run on the Canu draft genome before Nanopolish polishing, 19,970 errors were corrected, confirming that Nanopolish improves the draft genome.

**Figure 2.**
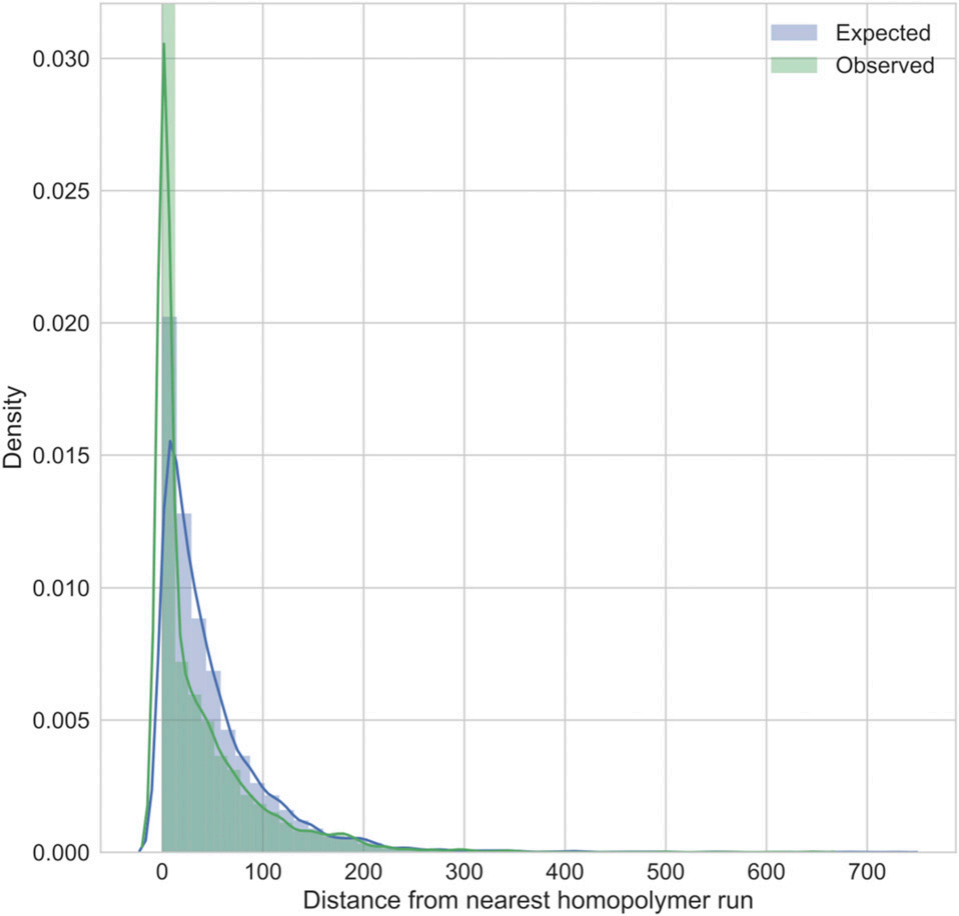
The observed distance from a homopolymer run for the errors corrected by Pilon in the draft genome, compared with the expected distribution if errors were randomly distributed across the genome. This figure appears in color at www.ajtmh.org.

We assessed the ONT read coverage and proximity to a homopolymer run (five bases or longer) for each of the errors (both single-nucleotide polymorphism [SNP] and indel) we corrected with Pilon. We observed no difference in the coverage distribution at sites with errors compared with the overall coverage distribution (Supplemental Figure 2). However, the positions around homopolymer runs were enriched for errors (two-tailed Kolmogorov–Smirnov test statistic=0.019, *P* = 0.035) (Figure 2). Whereas 15% of the final polished genome is within 5 bp of a homopolymer run (165 kb), 43% of errors (2,641) are within 5 bp of a homopolymer run.

The Illumina data alone were assembled using Unicycler to compare with assemblies using ONT only and the combined ONT and Illumina data. The assembly generated by Unicycler had 29 contigs, with a total length of 1,161,002 bp and an n50 of 166 kb. However, this included 20 short contigs under 6 kb, which are closely related to the mouse genome by BLAST search. These contigs are identified by their low coverage depth relative to *R. typhi* contigs. After removal of contaminating mouse contigs, the Unicycler assembly has eight contigs with a total length of 1,111,455 bp.

Furthermore, BUSCO was used to quantitatively measure the genome completeness of our assemblies based on a set of 48 conserved single-copy genes from the OrthoDB v9 database^33^ (Table 2). None of the genes were found in the initial Canu assembly. Inspection of the TBLASTN results shows that while there were hits to many of the conserved genes, they fall below the threshold where BUSCO will call them as present. After polishing with Nanopolish using ONT data only, 14.2% of the genes could be found as a complete copy, with a further 32.4% found as fragmented genes. Two rounds of Pilon polishing improve this to 88.5% of genes found as complete copies. Running BUSCO on the Wilmington reference strain gives the same number of complete genes, suggesting this is the complete set of the orthologous reference genes that exists in *R. typhi.* The Unicycler assembly also contains this complete set of genes.

**Table 2:** Proportion of BUSCO bacterial gene set found as complete genes, fragmented genes, or missing for different assemblies of the TM2540 strain, and the reference Wilmington assembly

	Complete (%)	Fragmented (%)	Missing (%)
Canu only	0.00	3.40	96.60
Canu + Nanopolish	14.20	32.40	53.40
Canu + Nanopolish + Pilon (1 round)	85.10	2.70	12.20
Canu + Nanopolish + Pilon (2 rounds)	88.50	0.70	10.80
Canu + Nanopolish + Pilon (3 rounds)	88.50	0.70	10.80
Canu + Nanopolish + Pilon (4 rounds)	88.50	0.70	10.80
Unicycler (Illumina only)	88.50	0.70	10.80
Reference genome (Wilmington)	88.50	0.70	10.80

All complete genes were single copy.

The final polished genome assembly is 1,111,939 bp, extremely similar to the three available genomes, which range from 1,111,496 to 1,112,957 bp in length (Table 1). Alignment of the four whole genomes in Mauve^34^ shows that the genomes are colinear and that no rearrangements have taken place between strains (Supplemental Figure 3).

The TM2540 genome contained 866 predicted genes, very close to the 865 genes predicted in all other strains. Roary was used to define the core genome (genes present in all strains at 90% identity) of the four *R. typhi* strains, which included 863 genes. Of the five accessory genes not found in all strains, two were found in three of the four strains and three were unique to a single strain. Two of the genes not found in all samples were the same gene (RT_RS01750 in strain Wilmington), but clustered separately because of a 150-bp insertion in the B991CWPP strain that caused the sequence to fall below the 90% identity threshold to cluster with the other three strains. In two of the five accessory genes, a single base insertion in one strain has created a premature stop codon, splitting a gene into two sequences in that strain (RT_RS02175 in strain Wilmington, and TM2540_RS03925 in strain TM2540). In the final case, the gene is present in three strains next to a duplicate pseudogene which has lost the start and end of the gene, whereas in the fourth strain, a deletion has removed the end of the pseudogene and the start of the gene, leaving two incomplete and presumably inactive copies of the gene (RT_RS03370 in strain Wilmington). All five variably present genes are annotated as hypothetical proteins in all strains.

SNPs and indels in the four genomes were called from the alignment of the four reference genomes. We found 32 SNPs and 30 small indels (size range 1–782 bp). A full listing of the variants and their location in the Wilmington strain genome is given in Supplemental Table 2. Table 3 shows the number of differences between strains (counting each indel as a single event).

**Table 3:** Pairwise differences between strains based on the whole genome alignment

	Wilmington	B9991CWPP	TH1527	TM2540
Wilmington	0			
B9991CWPP	27	0		
TH1527	18	21	0	
TM2540	40	45	36	0

One of the small indels is in the RARP2 gene, which was previously noted to have a variable number of ankyrin repeats. Our new strain TM2540 has the same variant previously found in B9991CWPP and TH1527 and differs from the reference Wilmington strain. We also note a deletion in penicillin-binding protein 4 (*pbpE)* in TM2540 which leads to a truncated protein product. Variants are also present in the surface cell antigen (sca) gene families, with SNPs in *sca1* and *sca3* and indels in *sca2* and *sca4*.

We compared the results of the final polished assembly to the best assemblies generated using Illumina data alone (filtered Unicycler assembly) or ONT data alone (Canu + Nanopolish assembly). The Illumina assembly has 866 predicted genes, and the core genome using this assembly has 862 core genes and seven accessory genes. This includes one gene unique to the Illumina assembly, which is part of a gene split into two pieces at a contig boundary. The ONT assembly has 1,745 predicted genes, but this number is inflated by a number of genes which have been split into multiple pieces likely because of sequence errors introducing premature stop codons in the sequence, and the core genome using this assembly has only 774 genes.

## DISCUSSION

WGS of pathogens is an increasingly important tool in both research and clinical settings, whose use has increased over the last decade as its cost has decreased and availability improved. However, the initial investment in sequencing and computing equipment and the technical expertise needed to produce and analyze data remain a barrier to its introduction into resource-poor settings. Although our research laboratory can perform molecular diagnostics and cell culture, to date there has been no sequencing capacity, necessitating the slow and costly shipment of samples to other countries to undertake sequencing projects. The portable nature of the MinION sequencer has allowed the first bacterial WGS to be performed in Laos. We were able to run all steps of our bioinformatic analysis (except Centrifuge) on a laptop computer (MacBook Pro 2017) without continuous Internet access, demonstrating that sequencing and analysis can take place in relatively remote settings.

Murine typhus is an important and severely neglected tropical and subtropical disease of worldwide distribution. In Vientiane, an estimated 10% of non-malarial fevers in adult inpatients are caused by *R. typhi.*^35^ Surprisingly, for a disease to which many millions of people are potentially exposed, there are only three published whole genome sequences, of which the last human isolate dates back more than 50 years.

The *R. typhi* genome is 1.1 Mb and contains almost no repeats, which makes genome assembly for this species relatively simple. Running Canu with the default ONT parameters was sufficient to assemble the genome into a single contig, and we polished the assemblies using both Nanopolish, to use the signal-level data from ONT sequencing, and Pilon, to use the extra information available from Illumina sequencing with its lower error rate. Although its synteny with other genomes suggests that there are no large misassemblies in our genome, the two methods of polishing corrected many small SNPs and indel errors in the ONT only draft genome. Thus, even for relatively simple bacterial genomes, a combination of ONT with data from another technology with a low error rate is currently necessary to produce an accurate sequence. In the case of *R. typhi*, the strains are so closely related that distinguishing between them requires a highly accurate sequence, and using ONT data alone, the sequencing errors are more numerous than the true differences between strains. Removing regions of the genome close to homopolymer runs would remove a large number of positions with errors by filtering out a comparatively small amount of the genome, but would still leave over 2,000 more errors than true differences. Oxford Nanopore Technologies data alone may suffice for applications which can work with sequences which still retain some base pair–level errors, such as determining large-scale genome rearrangements,^36^ determining the species of an unknown sample,^37,38^ or detecting antimicrobial resistance genes.^39^ Increases in sequencing accuracy, combined with bioinformatics advances in assembly and polishing software, may allow for the future use of ONT data alone to give complete and accurate sequences, increasing the applications for this device.

Comparative analysis of the four *R. typhi* genomes shows very little variation between strains. Very few genes are variably present between strains, and the gene order is completely conserved. Although these four samples were collected from humans and a rodent over a long timeframe, we see only 23 SNPs between the two most divergent strains, no large-scale genome rearrangements, and very few indels. The low number of differences suggests that WGS may not be a useful tool to analyze the relationships between strains of *R. typhi*, as there may be few differences between strains and no recent shared ancestry. Recent phylogenomic studies of multiple rickettsial species have identified very little divergence within species.^40,41^ Despite this small amount of variation, there are SNPs and indels which are private to each strain and could potentially form the basis of a molecular typing scheme to differentiate lineages of *R. typhi.* There are also variants in four of the five sca family genes present in *R. typhi*, which are important in adhesion and invasion of mammalian cells,^42^ and variation may be expected in these genes because of the selective pressure they are under as cell surface antigens; however, they are also large proteins and may be more likely to accumulate variants because of their size.

Some limitations remain on the use of ONT’s MinION platform. Currently, individual flow cells remain costly at USD500–USD900,^43^ depending on the number purchased. However, the cost per sample can be reduced because multiplex sequencing now allows for multiple bacterial strains to be sequenced on the same flow cell.^44^ Ongoing improvements in the technology continue to increase overall output and per-run capacity. Costs for setting up MinION sequencing facilities such as an appropriate laptop computer, shipping reagents, and flow cells are expected to be low. Shipping DNA or RNA at −80°C may cost more than USD1,000 per shipment and frequently requires supporting documentation and permissions. These costs remain a fraction of those required to set up and maintain non-portable sequencing platforms. Novel technologies are likely to improve access to sequencing when computational resources and support are limited and allow more cost-efficient sequencing of single samples.^45^

MinION instruments continue to be used in an ever-widening set of challenging circumstances. The platform has been used to detect and monitor nosocomial tuberculosis in Zambia,^46^ to sequence Ebola and Zika virus in Guinea and Brazil^3,47^ at field sites, to sequence amphibian DNA in montane rainforest in Tanzania,^48^ and for off-line use in the high Arctic and on the International Space Station.^49–51^ Here, we demonstrate the current applicability of the MinION to resource-poor settings where some laboratory infrastructure exists, but WGS capacity is unavailable. With concerns in some countries about the export of biological samples for WGS in other countries, MinION systems could facilitate countries without current WGS facilities to undertake such work.

## Supplemental tables and figures

Supplemental materials
