# Supplementary material for: Oxford Nanopore MinION Sequencing Enables Rapid Whole Genome Assembly of Rickettsia typhi in a Resource-Limited Setting
Source: Am J Trop Med Hyg. 2019 Dec 9;102(2):408–14. doi: 10.4269/ajtmh.19-0383 (PMC7008338; doi:10.4269/ajtmh.19-0383)
Supplement: Supplementary file 1 [file tpmd190383.SD1.pdf]

Supplementary Information

Figure 1. The coverage distribution for all positions in the genome, and positions where an error was corrected by Pilon.

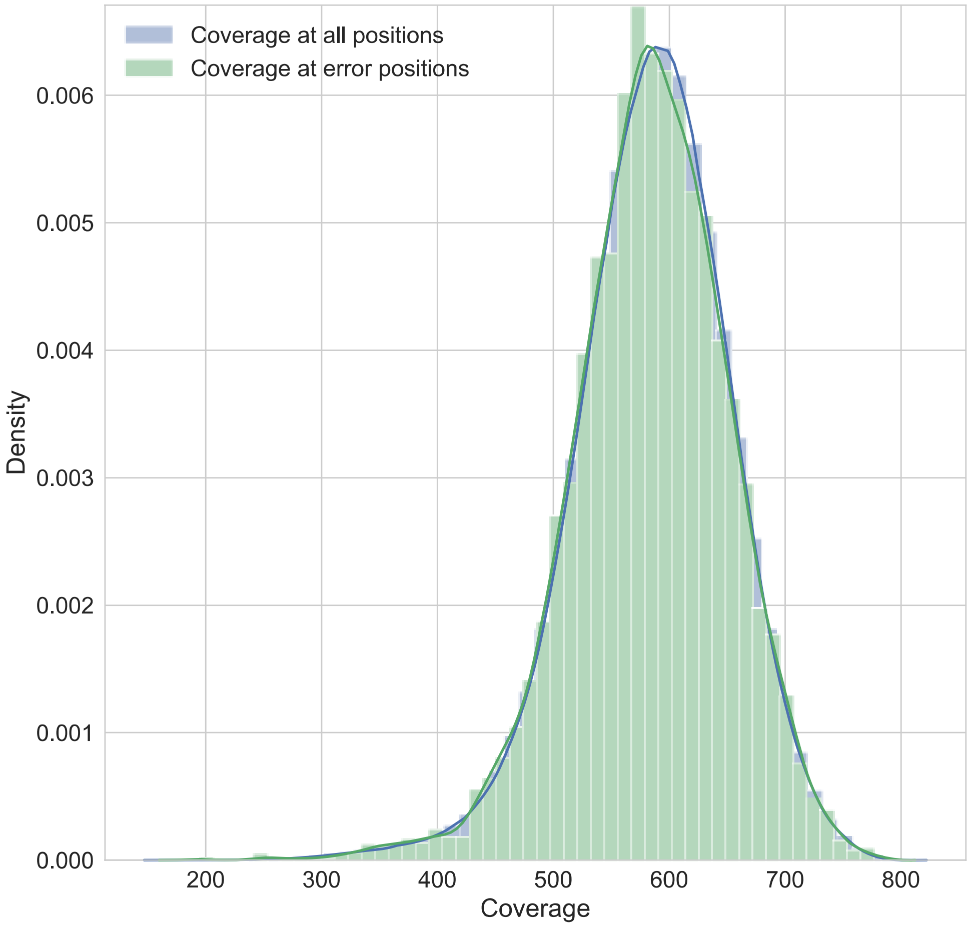

Table 1. The number of SNPs and indels which were corrected by successive rounds of polishing using Pilon.

	SNPs	Indels	Total
Round 1	125	5609	5734
Round 2	101	197	298
Round 3	0	48	48
Round 4	0	2	2

Figure 2. Alignment of the four strains using progressiveMauve. The genome alignment has one collinear block with no rearrangements.

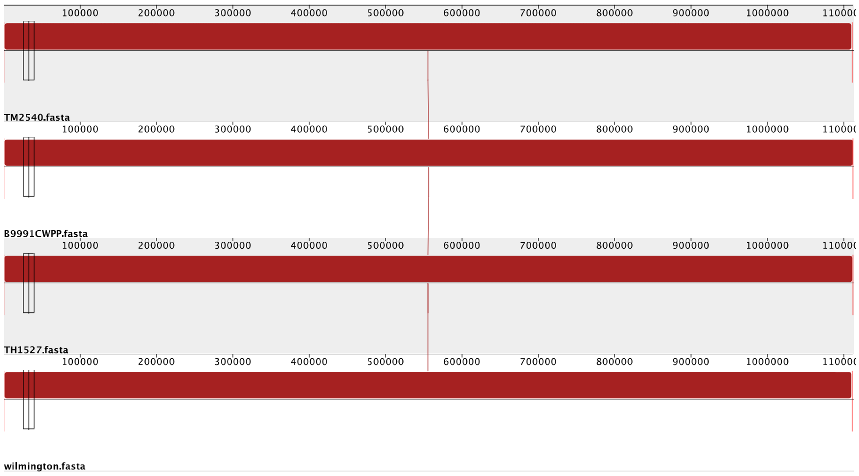

Table 2. List of variants found in the four R. typhi genomes. The Wilmington genome is used to determine the reference allele and the position of the variant, and marks the first base of the reference allele in the case of an insertion or deletion. Genotypes are given as 0 for the reference allele and 1 for the alternative allele. The locus tag, old locus tag and product are taken from the current version of the Wilmington genome found in RefSeq.

Note: Supplemental Tables 2 will be available online in final publication
